# Postoperative red cell distribution width to platelet ratio is related to cardiac surgery-associated acute kidney injury

**DOI:** 10.1186/s12872-025-05407-y

**Published:** 2026-02-23

**Authors:** Zhao-Xi Li, Chen-Yi Cui, Xiao-Liang Qian, Jia-Xin Huang, Jun-Long Hu, Bao-Cai Wang, Jian-Zhao Li, Zhao-Yun Cheng

**Affiliations:** 1 Department of Cardiac Surgery, Central China Fuwai Hospital, Zhengzhou University, Henan Provincial Clinical Research Center for Cardiovascular Diseases, Zhengzhou, China; 2 Department of Extracorporeal Circulation, Central China Fuwai Hospital, Zhengzhou University, Henan Provincial Clinical Research Center for Cardiovascular Diseases, Zhengzhou, China

**Keywords:** Red cell distribution width to platelet ratio, Cardiac surgery-associated acute kidney injury, Cardiopulmonary bypass, Inflammation

## Abstract

**Background:**

This study aimed to investigate the predictive value of the red cell distribution width-to-platelet ratio (RPR) for cardiac surgery-associated acute kidney injury (CSA-AKI).

**Methods:**

A retrospective analysis of clinical data from 252 patients undergoing cardiac surgery with cardiopulmonary bypass (CPB) was conducted. Patients were classified into AKI (*n* = 136) and non-AKI (*n* = 116) groups based on the Kidney Disease: Improving Global Outcomes (KDIGO) consensus criteria. Baseline creatinine was defined as the last measurement obtained before surgery. For the first seven days postoperatively, patients underwent sequential assessments of complete blood counts, hepatic function, and renal function. For the analysis, we used the laboratory values collected on the day immediately preceding the onset of AKI. Missing values were addressed using simple imputation. Continuous variables with an approximately normal distribution were imputed with their mean, whereas skewed variables were imputed using the median. Receiver operating characteristic (ROC) curve was used to determine the optimal cut-off value, and the area under the curve (AUC) was applied to compare predictive ability among different indices.

**Results:**

Clinical outcomes revealed significantly higher RPR levels in the AKI group compared to the non-AKI group (14.94 vs. 8.46, *p* < 0.001), with elevated RPR independently associated with AKI risk(Odd Ratio = 1.433, 95% CI: 1.158–1.774). The model satisfied the linearity-in-the-logit assumption, indicating that its estimated effects are reliable. ROC curve analysis demonstrated that RPR ranked second in predictive efficacy for CSA-AKI after blood urea nitrogen (BUN) (AUC = 0.855 vs. 0.926), with an optimal cutoff value of 11.416. Varieties’ combination analysis showed that combining RPR with BUN or C-reactive protein (CRP) significantly enhanced predictive accuracy, achieving an AUC of 0.978 for the RPR + CRP + BUN triad. The combined model was pre-specified prior to data analysis. The reliability had been verified and there is no interaction between variables. However, the study’s single-center design, inconsistent RPR measurement thresholds, and lack of external validation limited the generalizability of its findings. Thus, the design did not support establishing a causal link between RPR and CSA-AKI, necessitating validation through large-scale prospective trials. All ORs were subjected to adjustment. The AUC values were internally derived, with no external validation conducted.

**Conclusion:**

RPR may serve as a potential predictor for CSA-AKI, and its integration with conventional biomarkers could inform renal protection strategies.

## Introduction

Cardiac surgery on cardiopulmonary bypass (CPB) is strongly correlated with postoperative renal dysfunction, which represents one of the most frequent complications in this surgical population. This spectrum encompasses acute kidney injury (AKI), acute kidney disease (AKD), and chronic kidney disease (CKD), with AKI serving as a critical precursor to subsequent renal morbidity.^1^, ^2^ Before potential progression to AKD or CKD, AKI is independently associated with elevated short-term morbidity and mortality, as well as adverse long-term outcomes. Given its critical role in both early and late disease burden, cardiac surgery-associated acute kidney injury (CSA-AKI) has emerged as a priority research domain. CSA-AKI is operationally defined by the Kidney Disease: Improving Global Outcomes (KDIGO) consensus criteria as AKI occurring within seven days after cardiac surgery.^3^, ^4^ Its pathogenesis involves multifactorial mechanisms, prominently including CPB-induced systemic inflammatory responses. Distinct from conventional surgery, CPB induces multifaceted pathophysiological alterations encompassing extracorporeal oxygenation, systemic inflammation activation, and obligatory anticoagulation. CPB establishes a bloodless surgical field through cardiac arrest, enabling intricate intracardiac procedures. Blood contact with the extracorporeal circuit during CPB activates innate immune pathways through bio-incompatibility reactions with artificial surfaces, triggering the release of pro-inflammatory mediators.^5^ Concurrently, ischemia-reperfusion injury and oxidative stress synergistically potentiate inflammatory cascades, resulting in endothelial dysfunction and immune cell activation, which drives the systemic release of inflammatory cytokines including Tumor Necrosis Factor-α (TNF-α), Interleukin-6 (IL-6) and Interleukin-8 (IL-8) and complement activation products.^6^ Notably, reactive oxygen species (ROS) also induce transcriptional activation of pro-inflammatory pathways via nuclear factor kappa B (NF-κB) upregulation.^5^–^8^ The resultant microvascular dysregulation compromises renal hemodynamic autoregulation, while chemokine-mediated leukocyte infiltration promotes direct tubular injury and interstitial inflammation, ultimately culminating in the pathogenesis of AKI. Persistent inflammation may also contribute to progressive renal fibrosis through maladaptive repair mechanisms.^3^.

Red cell distribution width (RDW), a routine parameter in complete blood count (CBC), has evolved beyond its traditional role in anemia diagnosis and classification. Emerging evidence positions RDW as a biomarker of chronic inflammatory states, with elevated values reflecting underlying pathophysiological stress. Clinically, RDW demonstrates prognostic utility for cardiovascular morbidity, including hypertension progression, decompensated heart failure, atherosclerotic disease severity, and postoperative cerebrovascular events.^9^ Platelets (PLT), beyond their hemostatic functions, play a pivotal role in systemic inflammation, injury, and stress responses. Pro-inflammatory mediators such as IL-6 and IL-1β stimulate thrombopoiesis while enhancing platelet reactivity.^10^ The resultant thrombocytosis exacerbates microcirculatory dysfunction through multiple pathways: amplified platelet-leukocyte aggregate formation, increased vascular permeability, and perpetuation of pro-thrombotic inflammatory feedback loops. The red cell distribution width-to-platelet ratio (RPR), a novel composite biomarker integrating inflammatory and thrombotic dimensions, has demonstrated prognostic value in critical illnesses including septic shock and acute pancreatitis.^11^, ^12^ However, its association with CSA-AKI pathogenesis remains insufficiently characterized. This study is aimed at identifying the value of RPR for diagnosing CSA-AKI and evaluating the value of RPR combined with other factors for diagnosing CSA-AKI.

## Materials and methods

### Data source

This retrospective study that performed sample size calculation and met the requirement for the minimum sample size enrolled 252 consecutive patients who underwent cardiac surgery under CPB at Fuwai Central China Cardiovascular Hospital from January 2024 to December 2024. All enrolled patients were managed with a U.S.-imported Johnson & Johnson Cordis guide catheter (Model: MPD 6F 670-258−00) and a coated membrane oxygenator during CPB. Preoperative and early postoperative data were collected through medical record reviews and laboratory test results. Peripheral blood samples were obtained from all patients preoperatively and within seven days postoperatively for CBC, C-reactive protein (CRP), liver function, renal function, high sensitivity-cardiac troponin I (Hs-cTnI), N-terminal pro-B-type natriuretic peptide (NT-proBNP), and procalcitonin (PCT) analyses. All laboratory tests were performed according to standardized protocols in the Department of Laboratory Medicine. The patients’ last preoperative creatinine measurement was used as the baseline value. Changes in creatinine levels were monitored every 24 h and compared to this baseline to identify cases of CSA-AKI. For analysis, test results from the day before the CSA-AKI diagnosis were used, and the RPR was calculated based on the RDW and PLT values. We applied the Cleveland Clinic Score (CCS) by using patients’ preoperative data to calculate their respective scores. The CCS criteria was developed based on established findings in the literature and information sourced from MDCalc.^13^.

### Participant selection

In this study, initially 283 eligible participants were recruited. Following rigorous eligibility assessment, 15 patients were subsequently removed from the study due to incomplete postoperative follow-up data, eight patients exhibiting preoperative thrombocytopenia (defined as platelet count < 100 × 10⁹/L), two individuals receiving percutaneous coronary intervention (PCI) within seven days before CPB surgery and 6 non-survivors who died of multiorgan failure secondary to complications within the first postoperative week. Exclusion criteria included preoperative anemia (hemoglobin < 13 g/dL in males or < 12 g/dL in females), emergency surgery for acute myocardial infarction, thyroid disorders, autoimmune diseases, blood transfusion or donation within four months before surgery, history of surgical intervention within three months before surgery, preoperative thrombocytopenia (platelet count < 100 × 10^9^/L), and incomplete data. The primary outcome was postoperative AKI, defined by the KDIGO criteria. Patients were categorized into AKI and non-AKI groups based on these criteria. The final analytical cohort included 252 patients where 136 patients were diagnosed with CSA-AKI meeting the KDIGO criteria while 116 maintained normal postoperative renal function.

### Statistical analysis

For continuous variables with non-normal distributions, the Wilcoxon Mann–Whitney U test was employed and as medians with interquartile ranges and the median along with interquartile ranges was utilized as the relevant statistical measures. Means and standard deviations were used to summarize normally distributed continuous variables, while differences between the two groups were analyzed with Student’s t-test. Categorical variables were compared between two groups using Pearson’s chi-square test or Fisher’s exact test as appropriate for expected cell frequencies. The significance threshold for each test was set at less than 0.05. The association between RPR and postoperative AKI was evaluated using multivariable logistic regression analysis. Statistically significant variables were included in a logistic regression model with stepwise regression. Multicollinearity testing was employed to determine whether a strong linear correlation exists among the independent variables in the regression model. We conducted internal validation of the model using the bootstrap resampling method. And the calibration of the model was assessed using the Hosmer–Lemeshow test. Diagnostic performance was assessed through receiver operating characteristic (ROC) curve analysis, with the Youden index determining optimal cut-off values for investigated parameters. Predictive accuracy was quantified using the area under the ROC curve (AUC), complemented by calculations of sensitivity, specificity, positive predictive value (PPV), and negative predictive value (NPV). RPR was obtained by calculating the following equation: RPR, RDW(%)/platelet count (×10^4^/µL). PLR was calculated using the following formula: platelet count (×10^9^/L)/lymphocyte count (×10^9^/L). And LMR was derived from the formula below: lymphocyte count (×10⁹/L)/monocyte count (×10⁹/L). Because there were no missing values in the core dataset and the overall missing data rate was below 5%, simple imputation was applied. For continuous variables following a normal distribution, the mean value was used for imputation, while variables with a skewed distribution were imputed using the median. We verified the reliability of the model results using the likelihood ratio test (LRT). Furthermore, Our tests revealed that there is no interaction between variables. All these analyses were performed with the Statistical Package for the Social Sciences (SPSS) version 25 (IBM, Armonk, NY, USA). Figures were generated using SPSS, GraphPad Prism version 9 (GraphPad Software, San Diego, CA, USA), and R (R Foundation for Statistical Computing, Vienna, Austria).

## Results

### Baseline characteristics

**Fig. 1:**
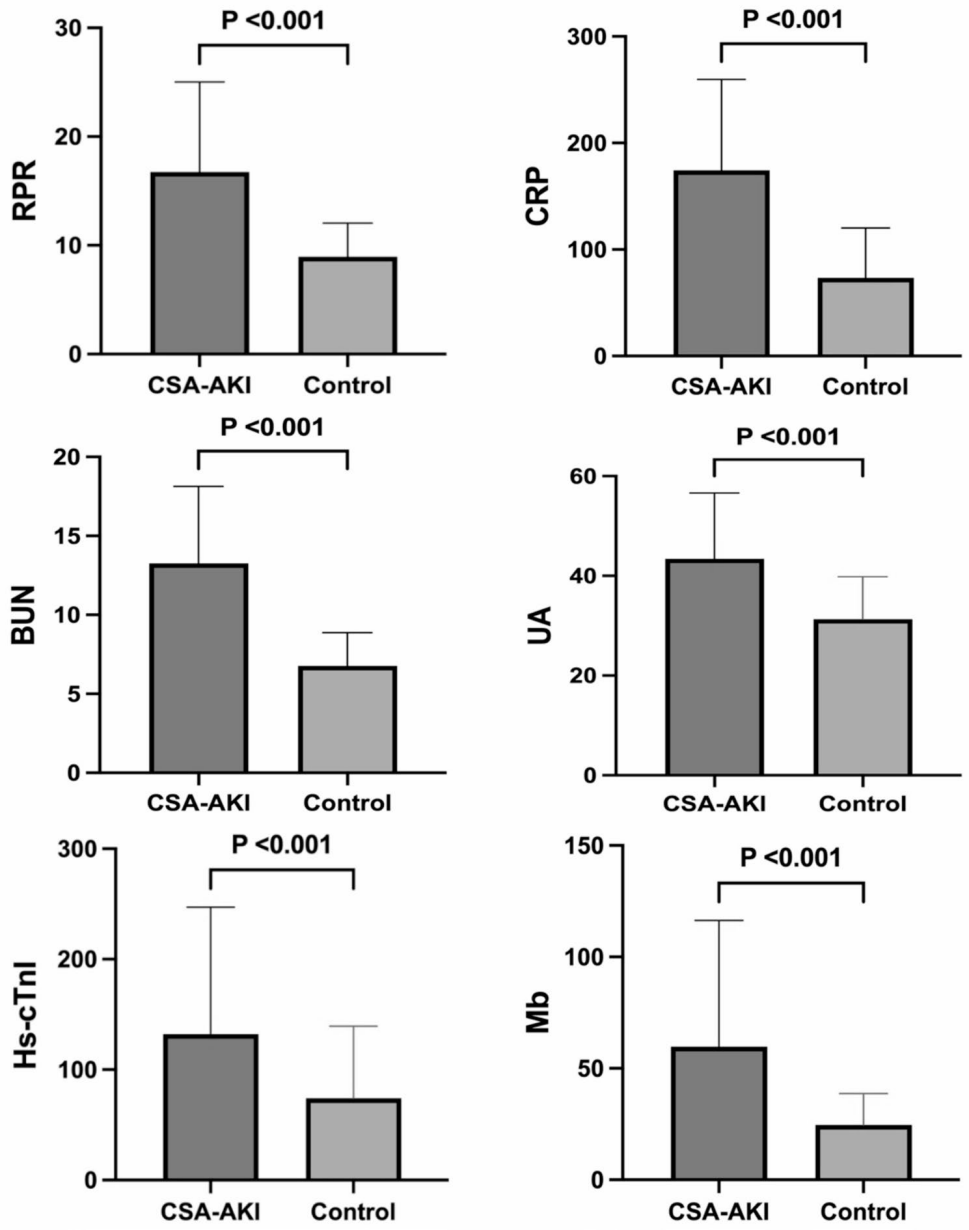
Differences in RPR, CRP, BUN, UA, Hs-cTnI and Mb levels between patients with CSA-AKI and control

The median age of the cohort was 70 years old (68,72). The median CPB duration and aortic cross-clamp time were 155.5 min (116, 204) and 98.5 min (65, 134.75) respectively (Table 1). Significant differences were revealed between the AKI and non-AKI groups: the AKI group exhibited higher RDW [14 (14, 15)% vs. 13 (13, 14)%; *p* < 0.001], lower platelet count [95 (73, 135.5)×10^9^/L vs. 159.5 (124, 201)×10^9^/L; *p* < 0.001] and elevated RPR (14.94 vs. 8.46, *p* < 0.001) (Table 1 and Table 2). Significant differences between groups were observed in multiple laboratory parameters, including CRP, total bilirubin (TBil), direct bilirubin (DBil), lactate dehydrogenase (LDH), blood urea nitrogen (BUN), uric acid (UA), anion gap (AG), creatinine (Cr), hemoglobin (Hb), and monocyte count (*p* < 0.001) (Table 4). Cardiovascular assessments demonstrated substantial disparities in key cardiac biomarkers. NT-proBNP, high-sensitivity cardiac troponin I (Hs-cTnI) and myoglobin (Mb) were all significantly higher in AKI group but creatine kinase-MB (CK-MB) became lower in this group (*p* < 0.001) (Fig. 1). In the AKI group, patients were categorized based on the KDIGO criteria: 107 were classified as Stage 1, 26 as Stage 2, and 3 as Stage 3 (Table 3).

**Table 1:** Comparison of demographic and clinical findings of patients classified by KDIGO criteria

Characteristics	All patients (n=252)	AKI (n=136)	No AKI (n=116)	*p*-value
Age (years)	70 (68, 72)	70 (68, 72)	69 (68, 72)	0.289
Male (n,%)	144 (57%)	81 (59%)	63 (54%)	0.401
Hypertension (n,%)	121 (48%)	64 (47%)	57 (49%)	0.742
Diabetes (n,%)	34 (13%)	15 (11%)	19 (16%)	0.216
Smoking (n,%)	65 (26%)	35 (26%)	30 (26%)	0.982
Alcohol (n,%)	49 (19%)	28 (21%)	21 (18%)	0.620
Hyperlipidemia (n,%)	121 (48%)	68 (50%)	53 (46%)	0.496
Weigh (kg)	65.35 (59.70, 72.30)	65.00 (58.92, 72.45)	65.80 (61.00, 72.15)	0.718
Aortic Clamping Time (min)	98.50 (65.00, 134.75)	115.50 (68.25, 154.75)	89.00 (58.00, 111.75)	<0.001
CPB Time (min)	155.50 (116.00, 204.00)	183.00 (133.00, 228.75)	134.50 (110.00, 168.00)	<0.001
Cleveland Clinic Score	3 (2,3)	3 (2,4)	3 (2,3)	0.019

**Table 2:** Summary of surgical procedures

Surgery type (*n*,%)	All patients (*n* = 252)	AKI (*n* = 136)	No AKI (*n* = 116)
CABG	23 (9%)	12 (9%)	11 (9%)
CABG + Mitral valve	43 (17%)	30 (22%)	13 (11%)
CABG + Aortic valve	23 (9%)	12 (9%)	11 (9%)
Mitral valve	78 (31%)	39 (29%)	39 (34%)
Aortic valve	23 (9%)	6 (4%)	17 (15%)
Mitral valve + Aortic valve	20 (8%)	13 (10%)	7 (6%)
Other	42 (17%)	24 (17%)	18 (16%)

**Table 3:** Stratified analysis by AKI severity

In-hospital AKI	Increase in Serum Creatinine	No. of cases *n*(%)
Stage 1	Increase ≥ 26.5 µmol/L (≥ 0.3 mg/dL) within 48 h, or 1.5–1.9 times baseline	107 (79%)
Stage 2	2.0–2.9 times baseline	26 (19%)
Stage 3	3.0 times baseline, or increase to ≥ 353.6 µmol/L (≥ 4.0 mg/dL), or initiation of renal replacement therapy (RRT)	3 (2%)

**Table 4:** Comparison of laboratory findings of patients classified by KDIGO criteria

Characteristics	All patients (*n* = 252)	AKI (*n* = 136)	No AKI (*n* = 116)	*p*-value
RDW (%)	14 (13, 14)	14 (14, 15)	13 (13, 14)	< 0.001
Platelet (×10^9^/L)	124 (89.25, 164.75)	95 (73.00, 133.50)	159.50 (124, 201.00)	< 0.001
RPR	11.11 (8.14, 15.85)	14.94 (11.13, 20.20)	8.46 (6.67, 10.72)	< 0.001
CRP (mg/L)	95.26 (56.99, 205.00)	181.16 (95.57, 253.76)	62.88 (43.52, 90.87)	< 0.001
Hb (g/dL)	109 (98, 119)	103 (95, 117)	112.50 (104.00, 120.00)	< 0.001
Lymphocyte (×10^9^/L)	0.63 (0.44, 0.92)	0.63 (0.43, 0.93)	0.63 (0.44, 0.91)	0.868
Monocyte (×10^9^/L)	0.81 (0.62, 1.07)	0.74 (0.55, 0.99)	0.92 (0.70, 1.12)	< 0.001
Neutrophil (×10^9^/L) PLR NLR LMR	12.11 (10.00, 15.36) 195.12 (126.75, 299.58) 19.40 (13.17, 29.28) 0.81 (0.57, 1.17)	11.99 (9.91, 15.70) 155.30 (97.47, 220.05) 19.37 (12.95, 29.18) 0.89 (0.58, 1.32)	12.30 (10.02, 15.25) 239.69 (164.37, 382.15) 19.62 (13.17, 29.48) 0.73 (0.51, 1.06)	0.626 < 0.001 0.785 0.006
ALT (U/L)	23 (16.10, 31.15)	23 (17.10, 32.75)	22.50 (16.00, 30.00)	0.373
AST (U/L)	63.50 (44.00, 86.75)	66 (44.25, 92.75)	61.25 (43.25, 83.00)	0.391
Total Protein (g/L)	62.14 ± 6.48	62.43 ± 7.33	61.80 ± 5.36	0.433
Albumin (g/L)	42.33 ± 3.66	42.59 ± 3.91	42.02 ± 3.33	0.220
Globulin (g/L)	19.82 ± 5.04	19.84 ± 5.15	19.79 ± 4.92	0.860
TBil (umol/L)	26.15 (15.93, 45.50)	30.70 (17.95, 54.55)	20.95 (14.30, 34.40)	< 0.001
DBil (umol/L)	14.85 (8.53, 26.78)	18.30 (10.88, 40.05)	11.10 (7.43, 19.35)	< 0.001
IBil (umol/L)	10.10 (6.73, 15.10)	10.60 (6.80, 16.28)	9.30 (6.63, 14.45)	0.430
LDH (U/L)	425.50 (365.00, 514.75)	461.50 (383.00, 540.75)	393 (350.50, 469.50)	< 0.001
BUN (mmol/L)	9.40 (6.53, 12.88)	12.25 (10.03, 15.48)	6.45 (5.30, 8.08)	< 0.001
Scr (umol/L)	105.50 (77.00, 142.50)	139 (119.25, 159.50)	77 (66.00, 85.00)	< 0.001
UA (mmol/L)	368.50 (297.00, 433.75)	410.50 (344.25, 511.50)	316.50 (257.25, 379.75)	< 0.001
NT-proBNP (pg/mL)	2265 (1272.50, 4152.50)	3129 (2114.50, 5329.00)	1451 (745.75, 2447.25)	< 0.001
Hs-cTnI (ng/mL)	684.15 (441.78, 1281.75)	1009.50 (570.38, 1566.75)	549.80 (345.03, 851.03)	< 0.001
Mb (ng/mL)	286.80 (176.73, 520.60)	446.70 (238.93, 804.98)	207.05 (153.00, 303.68)	< 0.001
CK-MB (ng/mL)	17.23 (8.84, 33.31)	11.80 (5.40, 26.02)	24.23 (14.87, 37.94)	< 0.001
AG (mmol/L)	16 (14, 18)	17 (15, 19)	15 (13, 17)	< 0.001
PCT (ng/mL)	2.34 (1.01, 7.00)	5.67 (2.19, 9.24)	1.06 (0.60, 2.10)	< 0.001

*RDW* Red cell distribution width, *RPR* Red cell distribution width to platelet ratio, *CRP* C-reactive protein, *Hb* Hemoglobin, *PLR* Platelet to lymphocyte ratio, *NLR* Neutrophil to lymphocyte ratio, *LMR* Lymphocyte to monocyte ratio, *ALT* Alanine aminotransferase, *AST* Aspartate aminotransferase, *TBil* Total bilirubin, *DBil* Direct bilirubin, *IBil* Indirect bilirubin, *LDH* Lactate dehydrogenase, *BUN* Blood urea nitrogen, *Scr* Serum creatinine,* UA* Uric acid, *NT-proBNP* N-terminal pro-B-type natriuretic peptide, *Hs-cTnI* High sensitivity-cardiac troponin I, Mb, myoglobin, *CK-MB* Creatine kinase-MB, *AG* Anion gap, *PCT* Procalcitonin

### Nonlinear relationship between RPR and CSA-AKI

All varieties exhibiting statistically significant associations in preliminary analyses were entered into a logistic regression model utilizing stepwise regression that identified 6 variables in the final results including RPR, CRP, BUN, UA, Hs-cTnI, and Mb. Multivariable logistic regression incorporating these variables revealed that elevated RPR was closely associated with AKI risk [Odd Ratio (OR) = 1.433, 95% Confidence Interval (95%CI) 1.158–1.774). The model further demonstrated that postoperative AKI was strongly related to CRP (OR = 1.037, 95% CI 1.020–1.054) and BUN (OR = 1.833, 95%CI 1.336–2.513). Additionally, UA, Hs-cTnI, and Mb exhibited statistically significant associations with renal injury outcomes. (Table 5) Assessment of multicollinearity revealed that the Variance Inflation Factor (VIF) of all variables was less than 5, indicating no multicollinearity in the model.

**Table 5:** Diagnostic performance of the tested varieties individually

Variables	OR (95% CI)	AUC (95% CI)	Youden index	Cut-off point	Sensitivity	Specificity	PPV	NPV
RPR	1.433 (1.158, 1.744)	0.855 (0.810, 0.901)	0.574	11.416	0.721	0.853	0.852	0.723
CRP	1.037 (1.020, 1.054)	0.831 (0.779, 0.883)	0.605	116.265	0.691	0.914	0.904	0.716
BUN	1.833 (1.336, 2.513)	0.926 (0.894, 0.957)	0.715	9.650	0.801	0.914	0.916	0.797
UA	1.164 (1.039, 1.303)	0.777 (0.721, 0.833)	0.409	37.050	0.676	0.733	0.748	0.659
Hs-cTnI	1.014 (1.005, 1.023)	0.708 (0.644, 0.772)	0.365	74.785	0.632	0.733	0.735	0.629
Mb	1.058 (1.012, 1.106)	0.763 (0.704, 0.823)	0.481	39.300	0.610	0.871	0.847	0.656

95% CI, 95% confidence interval, *AUC* Area under the curve, *PPV* Positive predictive value, *NPV* Negative predictive value, *RPR* Red cell distribution width to platelet ratio, *CRP* C-reactive protein* BUN* Blood urea nitrogen, *UA* Uric acid, *Hs-cTnI *High sensitivity-cardiac troponin I Mb myoglobin

### Predictive value of RPR and other variables

**Fig. 2:**
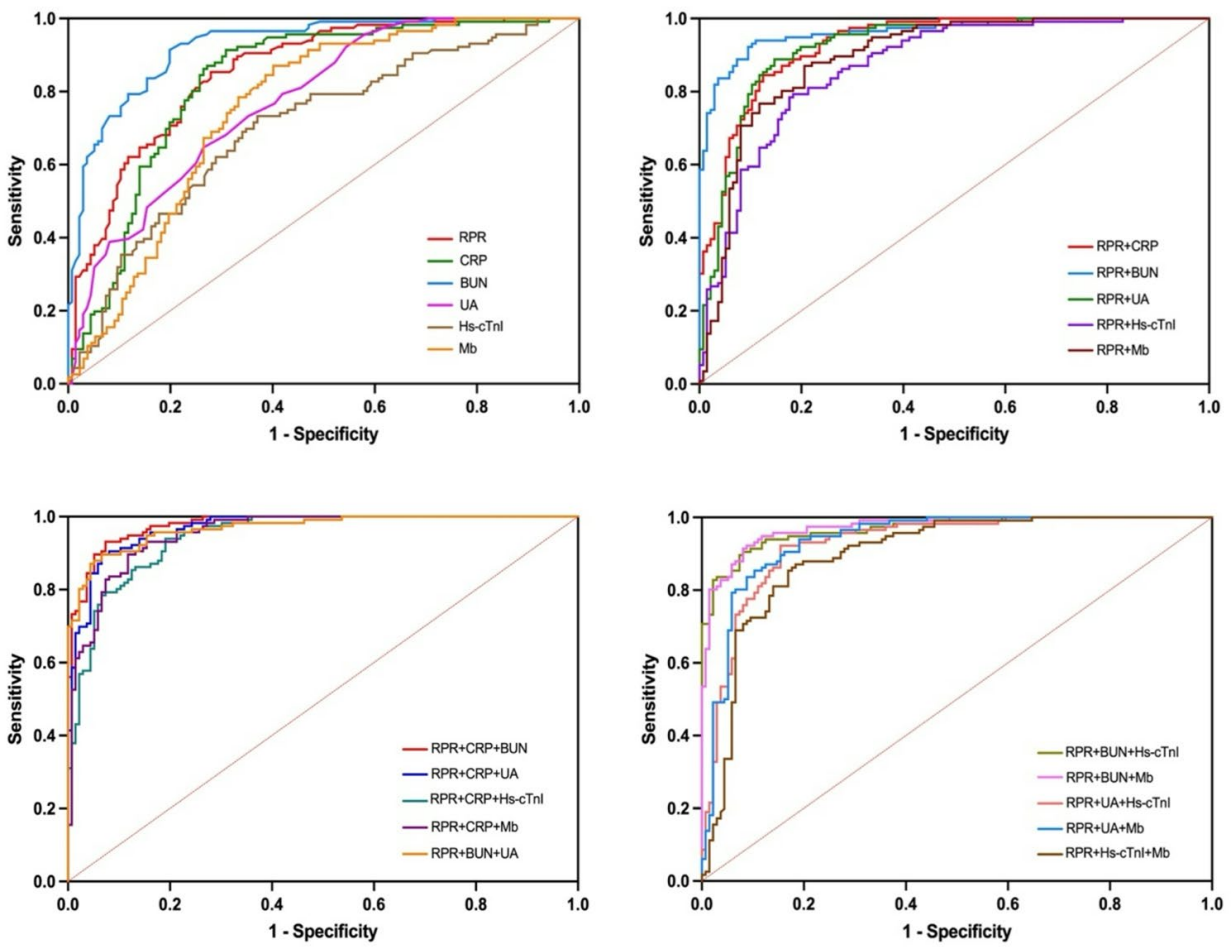
Receiver operating characteristic curves of RPR, CRP, BUN, UA, Hs-cTnI and Mb for predicting the incidence of cardiac surgery-associated acute kidney injury

**Fig. 3:**
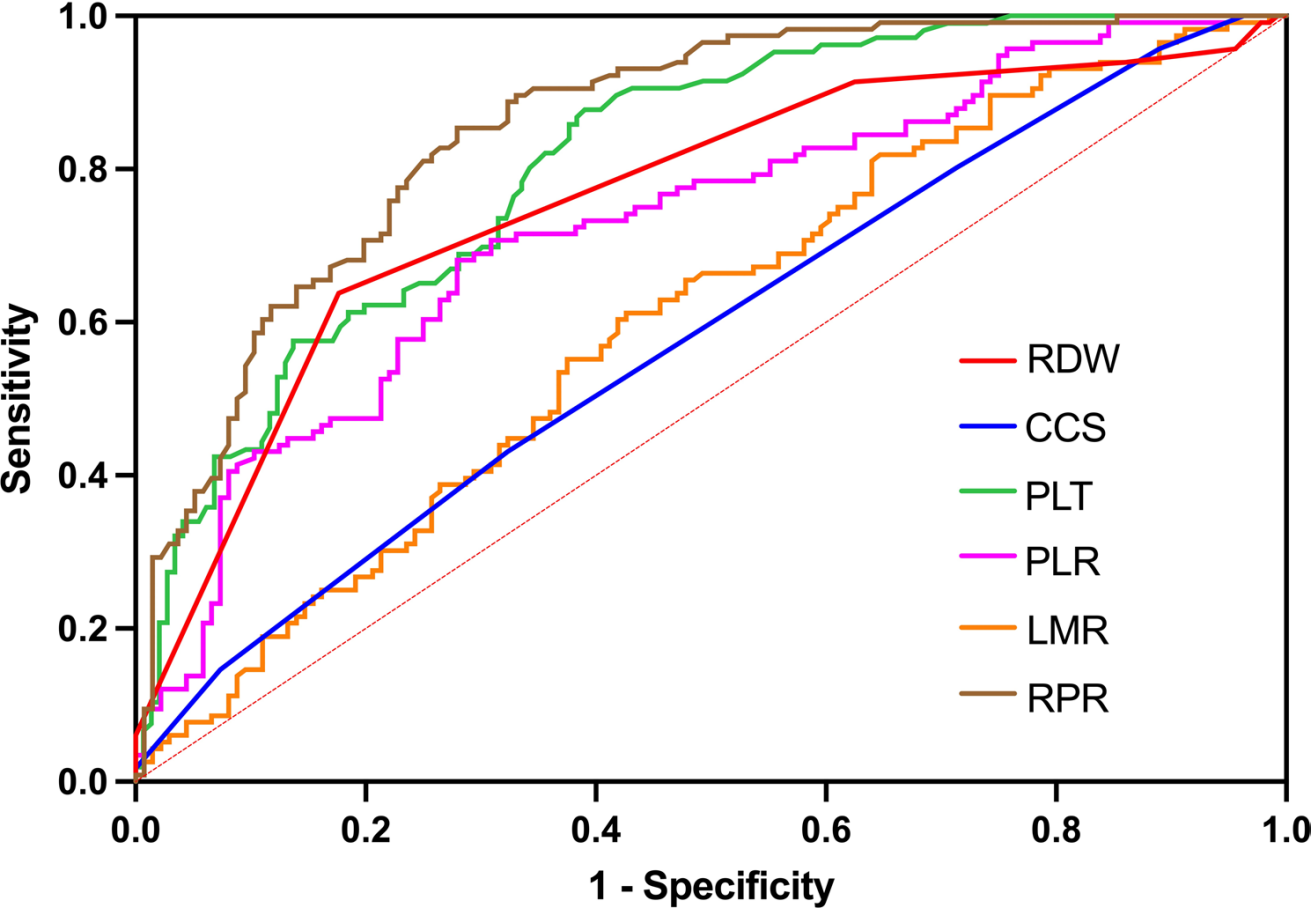
Comparison of ROC Curves among RPR, single indicators, other inflammatory markers, and scoring systems for predicting the incidence of cardiac surgery-associated acute kidney injury

ROC curve analysis identified that BUN exhibited the highest AUC of 0.926 (95% CI 0.894–0.957) differentiating CSA-AKI with an acceptable sensitivity of 0.801 and specificity of 0.914, using the optimal cutoff of 9.65 g/L determined by the Youden index for this study. RPR displayed robust discriminative capacity for AKI identification ranking the second AUC of 0.855 (95% CI 0.810–0.901). At its optimal cutoff of 11.416, the sensitivity is 0.721 and the specificity is 0.853. While demonstrating marginally reduced discriminative capability compared to BUN, CRP maintained clinically significant effectiveness in AKI identification achieving the third highest AUC of 0.831 (95%CI 0.779–0.883) showing the sensitivity of 0.619 and specificity of 0.914 at its optimal threshold of 116.265 mg/L. UA, Hs-cTnI, and Mb showed moderate predictive value but were less effective than the aforementioned markers. The integration of RPR with complementary biomarkers significantly augmented predictive performance for AKI. (Table 6) A two-marker panel combining RPR and CRP demonstrated superior diagnostic accuracy when the AUC was 0.931 (95% CI 0.901–0.960), achieving balanced sensitivity (0.875) and specificity (0.845) at an optimized threshold of 0.399. The combination of RPR and BUN outperformed all other pairwise varieties, attaining exceptional discriminative capacity to AKI (AUC = 0.964, 95% CI 0.943–0.985) with high concordance between sensitivity (0.890) and specificity (0.940) at the optimal cutoff of 0.594. The combination of RPR, CRP and BUN demonstrated highest diagnostic efficiency, achieving the AUC of 0.978. At an optimal cutoff value of 0.457, this triad exhibited balanced sensitivity of 0.926 and specificity of 0.931. The RPR + CRP + BUN combined model was established prior to data analysis. The results indicated that the model was well-calibrated. Furthermore, internal validation was performed, and the model was found to exhibit strong stability and generalizability. Notably, the integration of RPR with CRP and UA yielded the highest sensitivity of 0.934 whereas the combination of RPR, CRP, and Hs-cTnI achieved maximal specificity of 0.940. (Fig. 2) In addition, we have also compared the diagnostic efficacy of RPR with that of other single indicators and clinical scores. However, their AUC values were significantly lower than those of RPR (Table 7) (Fig. 3).

**Table 6:** Diagnostic performance of the tested varieties in combination

Combinations	AUC (95% CI)	Youden index	Cut-off point	Sensitivity	Specificity	PPV	NPV
Combination of two variables							
RPR + CRP	0.931 (0.901, 0.960)	0.720	0.399	0.875	0.845	0.869	0.852
RPR + BUN	0.964 (0.943, 0.985)	0.830	0.594	0.890	0.940	0.946	0.879
RPR + UA	0.922 (0.889, 0.956)	0.741	0.519	0.853	0.888	0.899	0.837
RPR + Hs-cTnI	0.869 (0.826, 0.913)	0.609	0.448	0.816	0.793	0.822	0.786
RPR + Mb	0.893 (0.853, 0.934)	0.665	0.527	0.794	0.871	0.878	0.783
Combination of three variables							
RPR + CRP + BUN	0.978 (0.965, 0.992)	0.857	0.457	0.926	0.931	0.940	0.915
RPR + CRP + UA	0.969 (0.951, 0.986)	0.831	0.432	0.934	0.897	0.914	0.921
RPR + CRP + Hs-cTnI	0.945 (0.919, 0.970)	0.749	0.679	0.809	0.940	0.941	0.808
RPR + CRP + Mb	0.953 (0.930, 0.977)	0.779	0.520	0.882	0.897	0.909	0.866
RPR + BUN + UA	0.969 (0.951, 0.987)	0.831	0.399	0.934	0.897	0.914	0.921
RPR + BUN + Hs-cTnI	0.967 (0.948, 0.986)	0.824	0.507	0.919	0.905	0.919	0.905
RPR + BUN + Mb	0.972 (0.956, 0.989)	0.834	0.533	0.912	0.922	0.932	0.899
RPR + UA + Hs-cTnI	0.927 (0.895, 0.960)	0.768	0.572	0.846	0.922	0.927	0.836
RPR + UA + Mb	0.938 (0.908, 0.967)	0.750	0.427	0.897	0.853	0.877	0.876
RPR + Hs-cTnI + Mb	0.897 (0.856, 0.937)	0.687	0.539	0.816	0.871	0.881	0.801

**Table 7:** Diagnostic performance of other biomarkers and clinical scoring system

Variables	OR (95% CI)	AUC (95% CI)	Youden index	Cut-off point	Sensitivity	Specificity
RPR	1.433 (1.158, 1.744)	0.855 (0.810, 0.901)	0.574	11.416	0.721	0.853
RDW	1.715 (1.051, 2.917)	0.762 (0.701, 0.822)	0.462	13.500	0.824	0.638
PLT	0.970 (0.954, 0.986)	0.830 (0.781, 0.879)	0.526	109.500	0.647	0.879
PLR	0.994 (0.992, 0.997)	0.726 (0.664, 0.789)	0.402	203.606	0.721	0.681
LMR	2.175 (1.296, 3.651)	0.601 (0.532, 0.671)	0.186	0.823	0.574	0.612
CCS	1.366 (1.088, 1.716)	0.582 (0.512, 0.653)	0.107	2.500	0.676	0.431

*PLR* Platelet to lymphocyte ratio, *NLR* Neutrophil to lymphocyte ratio,* LMR* Lymphocyte to monocyte ratio, *CCS* Cleveland Clinic Score

## Discussion

RPR has been demonstrated to correlate significantly with various infectious and cardiovascular disorders. This prospective cohort study investigated the association between preoperative RPR levels and postoperative AKI in 252 patients undergoing open-heart surgery with CPB. Our findings revealed a dose-dependent association between elevated RPR concentrations and increased AKI incidence. In predictive performance analyses, RPR exhibited superior diagnostic accuracy compared to CRP, UA, Hs-cTnI and Mb, though marginally lower than BUN. These findings collectively suggest RPR may serve as a promising predictor for CPB-associated AKI risk stratification. Notably, the integration of RPR with conventional varieties demonstrated augmented predictive efficacy, highlighting the clinical potential of RPR in perioperative renal protection strategies.

RDW has been established as a novel inflammatory prognosticator across diverse pathologies, including functional bowel disorders, autoimmune conditions, malignancies, and chronic diseases requiring recurrent hospitalization.^14^–^16^ Mechanistically, RDW was elevated because of AKI-induced oxidative stress, metabolic dysregulation, and hemodynamic instability which collectively enhance erythrocyte destruction rates, promoting immature reticulocyte release into circulation.^17^, ^18^ Platelets exacerbate inflammatory progression through pro-inflammatory cytokine secretion and immunocyte interactions.^19^, ^20^ Of clinical significance, thrombocytopenia in critically ill patients demonstrates strong correlation with adverse outcomes.^21^ RPR integrates these pathophysiological dynamics, emerging as a robust inflammatory indicator across multiple disease.^22^, ^23^ Clinically validated as a novel prognostic biomarker, RPR demonstrates predictive capacity for disease trajectories in neonatal sepsis, acute pancreatitis, and advanced hepatic fibrosis and hepatic cirrhosis.^11^, ^24^–^26^ Its clinical utility is enhanced by its routine accessibility through standard complete blood count parameters, non-invasive acquisition with rapid analytical turnaround, and superior diagnostic accuracy compared to conventional inflammatory markers.^23^, ^27^ These characteristics make RPR as a cost-effective prognostic tool with significant translational potential in resource-constrained settings. Compared with traditional indicators such as PLR, LMR, and CRP, RPR demonstrates markedly stronger predictive performance. Because the primary endpoint of the CCS is postoperative renal replacement therapy (RRT), its predictive accuracy is limited in settings where most patients experience only mild renal injury. In this research, the majority of cases involved AKI stage 1, which likely reduced the diagnostic performance of the CCS. A study published in *The New England Journal of Medicine* reported a similar distribution of CSA-AKI severity. In contrast to the CCS, both RPR and its composite indicators demonstrated stronger predictive value for identifying patients at risk of developing AKI.^28^.

RPR also plays an important role on treatment decisions. Beyond inflammatory responses, renal hypoperfusion plays a crucial role in the development of CSA-AKI. Such perfusion abnormalities not only lead to renal medullary hypoxia and tubular injury but also trigger the body’s compensatory response mechanisms.^29^–^31^ Previous research has demonstrated that amino acid infusion can activate the renal functional reserve, thereby providing a protective effect against renal damage caused by hypoperfusion.^32^ The potential of amino acid therapy has also been explored in cardiac surgery settings. A large, multinational, randomized, double-blind, placebo-controlled trial (The PROTECTION Trial) was conducted to evaluate the efficacy of amino acid infusion in preventing CSA-AKI. Results indicated that patients who received intravenous amino acids had a significantly lower incidence of CSA-AKI compared with those given a placebo.^28^, ^33^, ^34^.

However, the PROTECTION trial did not include measurements of newly identified AKI biomarkers. According to the KDIGO criteria, these biomarkers are not considered the gold standard for AKI diagnosis.^28^ Although incorporating biomarker data would not have influenced the trial’s primary outcomes, such indicators play a crucial role in the diagnosis and management of AKI. In contrast to serum creatinine—which typically rises only after the onset of CSA-AKI—biomarker levels can increase within hours, providing an early warning that facilitates timely intervention. Moreover, variations in biomarker levels can reflect the extent of renal injury in real time, help predict disease progression and long-term prognosis, and enable dynamic assessment of a patient’s condition. Finally, biomarker fluctuations can offer direct feedback on therapeutic efficacy, allowing clinicians to refine treatment strategies and avoid both overtreatment and undertreatment.

This investigation has several methodological constraints requiring critical consideration. First, the retrospective single-center design relying on electronic health records inherently limits generalizability, compounded by an underpowered sample size that increases susceptibility to selection bias. The single-center study design, variations in RPR detection thresholds, and absence of external validation collectively restrict the generalizability of RPR findings. Moreover, the study design does not allow for the establishment of a causal relationship between RPR and CSA-AKI. Then, the observational nature of data introduces unavoidable risks of measurement inaccuracies and residual confounding, particularly regarding undocumented perioperative variables. Finally, only creatinine criteria were used to diagnose the development of CSA-AKI. Since diuretics were initiated on the first postoperative day as part of perioperative care in our center, urine output of patients could not be effectively assessed after surgery. These limitations collectively constrain causal inference and external validity of our predictive models. Crucially, the diagnostic performance of RPR requires external validation through prospective multicenter trials employing standardized data collection protocols.

## Conclusions

An elevated postoperative RPR appears to be tentatively linked to a higher risk of developing CSA-AKI. Consequently, RPR may serve as a simple and accessible auxiliary biomarker for identifying patients undergoing CPB who are at increased risk. When combined with other biomarkers, RPR could provide additional insights to support clinical decision-making in this population. Nonetheless, these findings should be interpreted with caution, as they remain preliminary and are limited by the retrospective, single-center study design. Validation through larger, prospective, multicenter studies is necessary to confirm these results and establish the clinical applicability of RPR before it can be formally adopted into routine practice.

## Data Availability

The data that support the findings of this study are available upon reasonable request.

## Abbreviations

RPR: Red cell distribution width-to-platelet ratio
CSA-AKI: Cardiac surgery-associated acute kidney injury
CPB: Cardiopulmonary bypass
ROC curve: Receiver operating characteristic curve
AUC: Area under the curve
BUN: Blood urea nitrogen
CRP: C-reactive protein
AKI: Acute kidney injury
AKD: Acute kidney disease
CKD: Chronic kidney disease
KDIGO: Kidney Disease: Improving Global Outcomes
TNF-α: Tumor Necrosis Factor-α
IL-6: Interleukin-6
IL-8: Interleukin-8
CBC: Complete blood count
ROS: Reactive oxygen species
NF-κB: Nuclear factor kappa B
RDW: Red cell distribution width
PLT: Platelets
Hs-cTnI: High sensitivity-cardiac troponin I
NT-proBNP: N-terminal pro-B-type natriuretic peptide
PCT: Procalcitonin
Mb: Myoglobin
PCI: Percutaneous coronary intervention
PPV: Positive predictive value
NPV: Negative predictive value
TBil: Total bilirubin
DBil: Direct bilirubin
LDH: Lactate dehydrogenase
BUN: Blood urea nitrogen
UA: Uric acid
AG: Anion gap
Cr: Creatinine
Hb: Hemoglobin
